# Viscoelastic microfluidics: progress and challenges

**DOI:** 10.1038/s41378-020-00218-x

**Published:** 2020-12-14

**Authors:** Jian Zhou, Ian Papautsky

**Affiliations:** Department of Bioengineering, University of Illinois at Chicago, Chicago, IL 60607 USA

**Keywords:** Viscoelastic flow, Elastic and inertial force, Microfluidics, Numerical modeling, Particle separation and cell sorting, 3D focusing, Physics, Engineering

## Abstract

The manipulation of cells and particles suspended in viscoelastic fluids in microchannels has drawn increasing attention, in part due to the ability for single-stream three-dimensional focusing in simple channel geometries. Improvement in the understanding of non-Newtonian effects on particle dynamics has led to expanding exploration of focusing and sorting particles and cells using viscoelastic microfluidics. Multiple factors, such as the driving forces arising from fluid elasticity and inertia, the effect of fluid rheology, the physical properties of particles and cells, and channel geometry, actively interact and compete together to govern the intricate migration behavior of particles and cells in microchannels. Here, we review the viscoelastic fluid physics and the hydrodynamic forces in such flows and identify three pairs of competing forces/effects that collectively govern viscoelastic migration. We discuss migration dynamics, focusing positions, numerical simulations, and recent progress in viscoelastic microfluidic applications as well as the remaining challenges. Finally, we hope that an improved understanding of viscoelastic flows in microfluidics can lead to increased sophistication of microfluidic platforms in clinical diagnostics and biomedical research.

## Modeling microscale manipulation of biofluids

Insights into the dynamic behavior of biological fluids on the microscale are enabling more efficient analysis of cells, bacteria, and other small biological particles for research and clinical diagnostics. Blood, saliva and other biofluids have viscoelastic properties, which means that they exhibit both viscous and elastic behaviors depending on the forces to which they are subjected. These properties shape the migration behaviors of suspended bioparticles in unique ways. Jian Zhou and Ian Papautsky of the University of Illinois at Chicago have reviewed current progress in understanding the migration behaviors in such fluids within microfluidic devices. The authors discuss design principles that have enabled the development of microfluidic systems capable of separating and purifying cells, bacteria, and small vesicles from highly heterogeneous biological specimens, and highlight future challenges that need to be addressed.

## Introduction

The emergence of microfluidics has triggered an increased interest in biological and healthcare applications^1–3^ and fueled the development of approaches to the sorting and isolation of synthetic and biological microparticles, including beads^4,5^, cells^6,7^, bacteria^8,9^, and extracellular vesicles (EVs)^10,11^. Numerous active and passive platforms^12^ have been demonstrated for precise and high-throughput manipulation of such microparticles. Magnetic^13–15^, electrical^16–18^, acoustic^19–23^ and optical^24–26^ forces are the most common principles for active microfluidic devices. These platforms generally offer precise, on-demand control of spatial distribution but require control of the forces used as well as sophisticated device architecture. Conversely, passive microfluidic platforms rely on biophysical properties, such as the size, density, shape and deformability of cells or particles. Some of the most prominent passive techniques are deterministic lateral displacement (DLD)^27^, pinched flow fractionation (PFF)^28^, hydrodynamic filtration (HDF)^29^, cross-flow filtration (CFF)^30^, shear-induced diffusion (SID)^31–33^ and inertial microfluidics (iMF)^6,34–43^.

While the majority of microfluidic systems are aimed at biological and clinical applications, most operate based on Newtonian fluid behavior. This is because in these systems, biological samples are diluted 5–100× and thus are no longer non-Newtonian. However, most unmodified biological samples, such as blood^31^, saliva^44^ and cytoplasm^45^, are viscoelastic in nature, making separation of cellular components within them challenging. Viscoelastic fluids are non-Newtonian and are generally macromolecular or feature complex microstructures, giving rise to unique phenomena such as bread dough climbing up a rotating rod (Weissenberg effect^46^). Neutrally buoyant particles suspended in such fluids migrate laterally in confined shear flows^47^, subject to imbalanced normal stresses that are strongly dependent on particle size.

When viscoelastic migration met microfluidics nearly a decade ago^48^, it triggered burgeoning interest in both the fundamental investigations of particle dynamics and the applications using size-based lateral migration. The ability to fabricate long and narrow microchannels (the ratio of channel downstream length to its characteristic length such as hydraulic diameter can easily exceed 1000× in microfluidic channels) enables experimental observation and probing of particle dynamics in viscoelastic flows, which otherwise mostly relies on numerical simulations^47^. Recent investigations have experimentally confirmed many of the complex interactions predicted by simulations, such as the inward driving force due to fluid elasticity^48–51^, outward directing effect of shear thinning^52,53^, and particle motion following secondary flows in straight square channels^54,55^. Improvements in the understanding of particle dynamics have resulted in the expansion of applications based on viscoelastic migration, such as the focusing and separation of bioparticles in viscoelastic microflows. Recent manipulations of macromolecules such as DNA^56^ and separation of submicron exosomes^57^ in viscoelastic microchannels push the limits of passive microfluidics and expand the dynamic range of microfluidic separations even further.

In this review, we aim to discuss the progress in viscoelastic microfluidics, bridging the physics of conventional viscoelastic fluids and the characteristics of microfluidic flows toward a broad range of applications based on particle cross-stream migration. The increasing number of publications in this burgeoning field has led to several review papers that include discussion of particle manipulation in viscoelastic flows. Lu et al.^58^ and by Yuan et al.^59^ reviewed applications of cell focusing and separation in microfluidic devices, while D’Avino and colleagues^47,60^ discussed the current understanding of particle dynamics in viscoelastic fluid, but mainly in a broad context of non-Newtonian fluids. We hope that this review will offer a more practical understanding of viscoelastic microfluidics and their applications in cell separations. Thus, we first discuss the basic underlying principles of viscoelastic microfluidics, including fluid elasticity, inertia, shear thinning, particle blockage ratio and secondary flow, as they collectively govern the particle dynamics in microflows. We will then examine the properties of viscoelastic fluids and their implications for suspended particles in confined shear flows to better understand particle dynamics in microfluidic channels. Particle migration in pressure-driven microchannels will be covered in inertialess viscoelastic flows (Re ≪ 1) as well as in more complicated situations when fluid inertia is nonnegligible (Re ≥ 1). We then discuss the three pairs of competing phenomena that dominate particle migration and the effects of channel geometry and particle properties. We will also provide a brief introduction to computational models used for predicting particle dynamics in viscoelastic flows. Throughout the review, we include boxes that describe fundamental and practical aspects of viscoelastic fluids. Finally, we will conclude the review with illustrative experimental results and applications of viscoelastic microfluidics, as well as the remaining challenges and outlook.

## Viscoelastic fluids and suspended particles

Lateral migration of particles in viscoelastic flow stems from the interaction of these particles with their suspending viscoelastic fluid. Understanding fluid properties is thus critical to deciphering and predicting the migration of particles in viscoelastic flows. Although viscoelastic fluids are generally well studied on the macroscale, they are far less common in microfluidics. In this section, we discuss some of the remarkable behaviors of viscoelastic fluids, focusing on their differences from Newtonian fluids and their properties (e.g., first and second normal stress differences) that are behind such differences and are responsible for particle migration in viscoelastic flows.

### Viscoelastic fluids

Viscoelastic fluids exhibit many differences in behavior from Newtonian fluids, such as the Weissenberg rod climbing effect^46^, which can occur even without rods^61,62^. Other pronounced phenomena in viscoelastic flows include extrudate swell^62,63^, large vortices and pressure drop when entering contraction geometry^63,64^, and melt fracture^46,62^. In microscale flows, viscoelastic fluids have been reported to exhibit (1) vortex formation at small Reynolds numbers (Re < 0.1) in a contraction microchannel^65^, (2) a longer required entrance length for full development of parabolic velocity profile and concave velocity profile observed during flow development^66^, (3) steady asymmetric flow patterns and unsteady 3D flow patterns in a T-junction microchannel^67^, and (4) suppression of vortex formation downstream obstructions in microchannels^68^.

The distinct behavior of viscoelastic fluids is attributed to their unique rheological properties (see Box 1). These fluids consist of both viscous and elastic components and thus behave like a viscous fluid in some circumstances and as an elastic solid in others^46,63^. Unlike the constant viscosity in Newtonian fluids (*η*, only dependent on temperature), the viscosity of viscoelastic fluids is typically a decreasing function of the shear rate due to the macromolecular nature^62^. This phenomenon is known as shear thinning and is found to drive outward migration of particles in viscoelastic flow^52,69^. The elastic behavior in these fluids is related to the normal stresses that are generated by the orientation and alignment of macromolecules along the flow direction^63,70^. It is the difference between these normal stresses that causes particle cross-stream migration in viscoelastic flow.

**Fig. 1:**
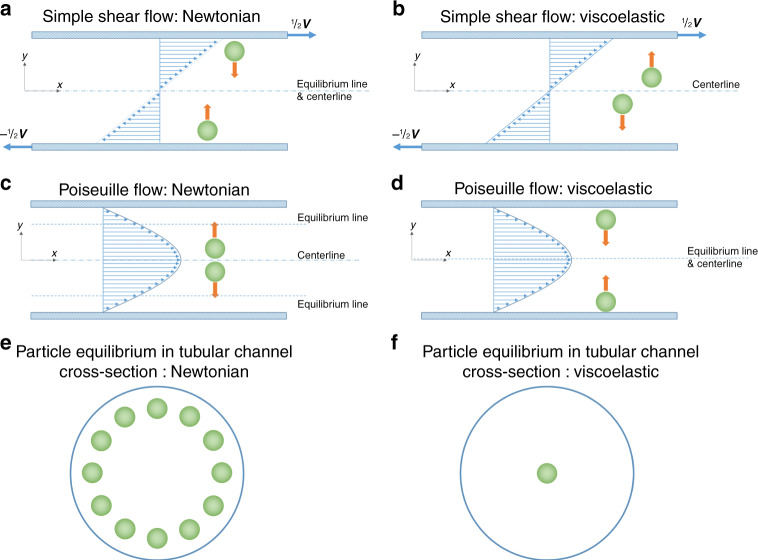
Illustrations of particle dynamics in simple shear flow and Poiseuille flow. In the case of simple shear flow, **a** particles migrate toward equilibrium positions at the centerline in Newtonian fluid dominated by inertial force^86,87^ but **b** migrate toward the walls in viscoelastic fluid^84,85^, regardless of particle initial position. **c** Particles laterally migrate to equilibrium positions near walls in Newtonian Poiseuille flow under the control of inertial forces^86^. **d** Particle laterally migrates to the centerline in viscoelastic Poiseuille flow undergoing elastic force^47,69^. **e** Particles equilibrate into an annulus near the sidewall of the circular channel in Newtonian flow undergoing inertial force. **f** Particles equilibrate into the center of the circular channel in viscoelastic flow dominated by elastic force

The normal stresses in viscoelastic flow are unequal, with two independent normal stress differences arising from the three normal stress components^62^. The first normal stress difference (*N*_1_) is defined as the difference between the streamwise normal stress (*σ*_*xx*_) and the transverse normal stress (*σ*_*yy*_)^62,70^. The coordinate system is set such that *x* is the downstream flow direction and *y* is in the direction of the gap (Fig. 1). Thus, *N*_1_ = *σ*_*xx*_ – *σ*_*yy*_, which gives rise to the material property designated as the first normal stress difference coefficient: ${\Psi}_1 = N_1/\dot \gamma ^2$, where $\dot \gamma$ is the shear rate^70,71^. In a Newtonian fluid, *N*_1_ is zero, as normal stresses are the same in all directions. In viscoelastic fluids, *N*_1_ accounts for many aforementioned phenomena, including the Weissenberg rod climbing effect^62^, and it is one of the major forces responsible for particle migration in viscoelastic flows^48^.

The other normal stress difference (*N*_2_) is defined as the difference between the transverse normal stresses (*N*_2_ = *σ*_*yy*_ – *σ*_*zz*_), which can be viewed as a measure of the relative stretching of macromolecules in the velocity gradient direction (*y* direction) versus that in the *z* direction^70^. Similar to *N*_1_, *N*_2_ gives rise to another material property as the second normal stress difference coefficient: ${\Psi}_2 = N_2/\dot \gamma ^2$. Coefficients ψ_2_ and ψ_2_, together with *η*, are the viscometric functions that fully characterize a viscoelastic fluid in simple shear^62,70^. Although *N*_2_ is approximately an order of magnitude smaller than *N*_1_ and is much more difficult to measure^62,72^, it is responsible for the occurrence of secondary flow orthogonal to the axis flow in noncircular channels^73–77^. This secondary flow can impact particle focusing in viscoelastic flow^54,55^, which will be revisited in detail in the section “Particle migration in viscoelastic microfluidics”.

Additionally, fluid relaxation time (*λ*) is a key parameter required to evaluate the effect of fluid elasticity on particle migration in viscoelastic flow. The relaxation time is the characteristic time required for macromolecules of the fluid to relax from a deformed state to their equilibrium configuration^78^, and it cannot be directly measured^79^. There could be a spectrum of characteristic times in a real-world fluid^79,80^. In Newtonian fluids, *λ* = 0, as they do not exhibit any time-dependent effects^70^. A common method to derive the relaxation time is through a separate evaluation of fluid viscous and elastic responses to a shearing sinusoidal deformation, followed by observations of the intersection of the loss and the storage moduli^79,81^. Indirect determination of the relaxation time was also reported based on the particle migration in the microfluidic channel^79^.

Box 1. Newtonian and viscoelastic fluids in the context of particle migration Viscoelastic fluids exhibit many differences in behavior from Newtonian fluids, such as the Weissenberg rod climbing effect^46^. The distinct behavior of viscoelastic fluids is attributed to their unique rheological properties. These fluids consist of both viscous and elastic components, and thus behave as viscous fluid in some circumstances and as elastic solid in others^46,63^. While in Newtonian fluids the viscosity (*η*) is considered constant at any given temperature, in viscoelastic fluids viscosity is commonly a decreasing function of shear rate. This is known as shear thinning and is due to the macromolecular nature of viscoelastic fluids^62^. It is shear thinning that drives outward migration of particles in viscoelastic flow (see Box 3 for details)^52,69^.

The normal stresses in viscoelastic flow are unequal, which differs from Newtonian flows. Two independent normal stress differences (*N*_1_ and *N*_2_) can be formed from the three normal stress components^62^. The elastic force that drives particle lateral migration in viscoelastic flow mainly arises from the first normal stress difference (*N*_1_). *N*_2_ is known to cause secondary flow in noncircular cross-sections of microchannels, which tends to disperse (defocus) particles. A parameter named fluid relaxation time (*λ*) is required to evaluate the effect of fluid elasticity on particle migration in viscoelastic flow. This is the characteristic time required for the macromolecules of the fluid to relax from deformed state to their equilibrium configuration^78^. Key properties of viscoelastic and Newtonian fluids regarding particle migration are summarized in the table below.

**Table:** 

	Newtonian fluid	Viscoelastic fluid
Material function	*η*	*η*, ψ_1_, ψ_2_
Normal stress differences	None	*N*_1_*, N*_2_
Shear thinning	No	Yes (commonly)
Relaxation time (*λ*)	*λ* = 0	*λ* *>* 0
Driving factors responsible for particle lateral migration	Inertial force, drag force	Elastic force (*N*_1_), inertial force, shear thinning, *N*_2_-induced secondary flow, drag force

### Particles in viscoelastic fluid

Particles suspended in viscoelastic fluid migrate across the streamlines due to the imbalance of normal stresses. Both numerical simulations^49,82–84^ and experimental results^84,85^ have shown migration of particles driven by the fluid viscoelasticity in a simple shear flow (planar Couette flow). Particles near the centerplane migrate toward the closest wall, which is opposite of particle migration in Newtonian fluid (Fig. 1a, b)^86,87^. Particles migrate rapidly toward the wall before an abrupt decrease in migration velocity^82,83^. Normal stresses are responsible for the cross-streamline motion, and no migration was found in a purely viscous fluid when inertia is negligible (Re ≪ 1; see Box 2 for dimensionless numbers)^83^. Furthermore, wall confinement is necessary for migration^83^.

In planar Poiseuille flow, where curvature of velocity profile and shear rate gradient exist, the elastic force (*F*_e_) stemming from the first normal stress difference (*N*_1_) drives particles toward the centerline where shear rate is lowest. This driving force depends strongly on particle size (*F*_e_ ~ *a*^3^). In contrast, fluid shear thinning forces particles to migrate toward the region of high-shear rate at the closest walls. The competition between the two factors suggests two stable equilibrium regions in the centerline and near wall. Lateral migration velocity, which is strongly dependent on particle size and fluid rheology, will also be discussed in this section.

Particle lateral migration has also been predicted and observed in Poiseuille flows (e.g., channel flow), which is more practical in the real world (Fig. 1c)^69,88–91^. Particle motion implicated by the imbalanced normal stresses is toward the low-shear-rate region within the flow^47,88^. In channel flow, the velocity gradient is not constant, and thus, the shear rate varies across the channel cross-section with the lowest in the channel centerline in a tube or in the central plane in a wide slit. As a result, particles are driven to the channel centerline or the centerplane under the control of fluid viscoelasticity. Tehrani^91^ confirmed that both the elastic property of the suspending fluid and the shear rate gradient are critical to the particle transverse motion. Despite the strong elasticity of the fluid, little migration in the flow with a central plug region or slip at the wall was observed in that work^91^.

When shear thinning of the viscoelastic fluid becomes relevant, particles are subjected to an additional tendency of moving to the high-shear rate region. Shear thinning causes particle migration in the opposite direction due to fluid elasticity. In channel flows, particles migrate toward channel walls instead of channel centerline if the shear-thinning effect is strong^52,69^. The migration behavior of particles is thus determined by the competing effects of fluid shear thinning and elasticity (Re ≪ 1). When shear thinning is excessively strong, particles migrate to the channel wall only; conversely, particles migrate to the channel centerline if elasticity is dominant^47,69^, such as in Boger fluids (elastic with constant viscosity)^92^. Sometimes, the term separatrix is employed to describe such competing effects on particle migration^47,93^. The opposing effects of fluid elasticity and shear thinning have been confirmed by direct numerical simulations in plane Poiseuille flow^49,94^ and demonstrated experimentally using polyvinyl pyrrolidone (PVP) and polyethylene oxide (PEO) solutions^48,95^. Nevertheless, shear thinning alone is insufficient to induce lateral displacement^47^. The simulation results by Huang and Joseph^52^ suggest that the shear-thinning effect is small when the inertia or shear rate is small.

Particle migration stemming from fluid viscoelasticity is strongly dependent on particle size^48,91^. Since the second normal stress difference (*N*_2_) is considerably smaller than *N*_1_, the driving elastic force (*F*_e_) of particle migration can be assumed to mainly depend on *N*_1_. This elastic force is in the direction of the low-shear-rate region (e.g., channel center) and scales with the normal stress gradient as *F*_e_ ~ *a*^3^∇*N*_1_^48,91^, where *a* is the particle diameter. By balancing this force with the Stokes drag (*F*_*D*_ = *6πaηV*) when shear thinning is negligible, the migration velocity (*V*) can be expressed as $V\sim \frac{{a^2}}{\eta }\nabla N_1.$ Further derivation^51,96^ shows that the migration velocity scales as

1 $$V\sim {\mathrm{Wi}}\,a^2\nabla \dot \gamma$$

Consequently, the particle migration scales with the square of particle size and is dependent on the fluid rheological properties.

A similar expression considering both first and second normal stress differences shows dependence of the migration velocity on the particle size and fluid viscoelasticity. At a small Deborah number (e.g., De < 0.1) and small blockage ratio (e.g., *β* < 0.12; see Box 2 for dimensionless numbers), a dimensionless migration velocity (*V*_M_), which is the ratio of local migration velocity (*V*) to the mean flow velocity in the main flow (*V*_M_ = *V*_*r*_/*V*), can be derived as^88–90,95,97^

2 $$V_{\mathrm M}\sim {\mathrm {De}}(1 + C\frac{{{\mathrm{{\Psi}}}_{2,0}}}{{{\mathrm{{\Psi}}}_{1,0}}})\beta ^2\frac{y}{H}$$

where ψ_1,0_ and ψ_2,0_ are respectively the first and second normal stress difference coefficients at zero shear rate, *C* ≅ 2, *H* is the low aspect ratio channel height and *y* is the vertical particle position (*y* = 0 is the central plane)^47,88^. Note that at large *β*, this expression may not hold, as Villone et al.^93^ found that all particles migrated toward the wall when *β* > 0.7. Although the second normal stress difference is generally small compared to *N*_1_, it induces recirculation orthogonal to the main flow in noncircular straight channels^54,73–77,98–100^. The intensity of such secondary flows in the channel cross-section is 2−3 orders of magnitude smaller than that of the main flow, but these flows may affect the particle migration^54,55^. The influence of the secondary flow will be revisited later in the context of microfluidic channel flow.

In this section, we have seen how viscoelastic fluids differ from classic Newtonian fluids. These differences are highlighted in Box 1. The normal stresses of viscoelastic fluids generate a set of complex viscometric parameters that fully describe these fluids in simple shear. These fluids and their flows can be characterized by nondimensional parameters, as shown in Box 2. All these can be directly applied to particles or cells suspended in such fluids. In the next section, we examine the impact of fluid viscoelasticity on particle migration.

Box 2 Dimensionless numbers in viscoelastic flows A group of nondimensional parameters used to characterize hydrodynamics and viscoelasticity of flows are summarized below. In addition to the commonly used Reynolds number (Re), the Deborah number (De) and the Weissenberg number (Wi) are used to characterize viscoelasticity of non-Newtonian flows. The former is the ratio of the fluid relaxation time to the time of observation (also denoted as fluid characteristic time); it describes the extent to which the response of a material to a deformation is viscoelastic rather than purely viscous^181^. The latter describes nonlinearity of rheological response and is the ratio of the elastic force (first normal stress difference) to the viscous force (viscous stress)^47^. For a given geometry, Wi and De are proportional to each other^181^ and are interchangeably used in some cases^83,95,176^. Note that both quantities are zero for Newtonian fluid as *λ* = 0. The elasticity number (El) indicates the relative importance of the elastic and inertial forces in shear flow^46^ and only depends on the fluid rheological properties and the characteristic length scale. When El ≫ 1, the fluid elastic force dominates, while the inertial stress is dominant when El ≪ 1. The two forces are considered comparable when ${\mathrm {El}} = {\cal{O}}(1)$^46^. Additionally, blockage ratio is the ratio of particle size and channel characteristic length.

**Table:** 

Dimensionless parameter	Symbol	Definition	Expression
Reynolds number^182^	Re	Ratio of inertial to viscous forces	${\mathrm {Re}} = \frac{{\rho VD_h}}{\eta }$
Weissenberg number^47,181^	Wi	Ratio of first normal stress to shear stress/ratio of elastic to viscous force	${\mathrm {Wi}} = \frac{{N_1}}{\tau } = {\uplambda}\dot \gamma$
Deborah number^47,181^	De	Ratio of fluid relaxation time to flow characteristic time	${\mathrm {De}} = \frac{\lambda }{{t_c}}$
Elasticity number^46,144^	El	Ratio of Weissenberg number to Reynolds number/ratio of elastic over inertial forces	${\mathrm {El}} = \frac{{{\mathrm {Wi}}}}{{{\mathrm {Re}}}} = \frac{{\lambda \eta (W\, + \,H)}}{{\rho W^2H}}$
Blockage ratio^47^	*β*	Ratio between characteristic lengths of particle and channel	$\beta = \frac{a}{{D_{\mathrm {h}}}}$

*ρ* fluid density, *V* average flow velocity, *D*_h_ hydraulic diameter, *D*_h_ = 2*WH*/(*W* + *H*), where *W* and *H* are the channel width and height, respectively, *η* fluid dynamic viscosity, *N*_1_ first normal stress difference, *λ* fluid relaxation time, $\dot \gamma$ shear rate, *t*_c_ flow characteristic time, *a* particle diameter

## Particle migration in viscoelastic microfluidics

**Fig. 2:**
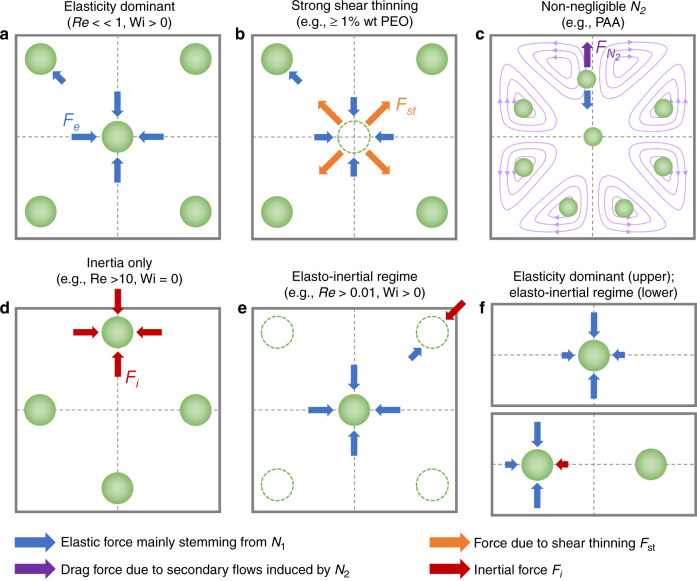
Particle migration and focusing governed by forces stemming from fluid elasticity, inertia, shear thinning, and their interactions in channels with square and rectangular cross sections. **a** Five focusing positions dominated by elastic force (*F*_e_) in inertialess viscoelastic flows without the shear thinning effect (e.g., Re < 0.01 and Wi > 0). **b** Shear thinning of fluid leading to defocusing of particles in the center and leaving four focusing positions near the corners. Here, *F*_st_ represents the effect of shear thinning driving particles toward the walls. **c** Strong secondary flows induced by the second normal stress difference (*N*_2_) causing dispersion of small particles. Here, *F*_*N*2_ represents the drag force acting on particles due to secondary flows. The dispersion of particles due to secondary flows induced by *N*_2_ was reported experimentally^55^. However, positions in the centers of vortices were suggested only in simulations^54^ and have not been experimentally observed. **d** Four focusing positions resulted from the balance of inertial forces (grouped as *F*_i_) in Newtonian inert_*i*_al flows (e.g., 10 ≤ Re ≤ 100 and Wi = 0). **e** Interaction of elastic force (*F*_e_) and inertial force (*F*_i_) leading to elimination of the corner positions in viscoelastic flows with nonnegligible inertia (e.g., Re > 0.01 and Wi > 0). **f** Single focusing position and two positions in rectangular channels depending on the interaction of elastic and inertial forces. When the inertia is small (e.g., low flow rate), a single focusing position is present in the center; when the flow rate increases, two positions emerge as inertia becomes relevant

Particle migration in microfluidic channels has been exploited for flow focusing and sorting devices in the past decade due to the strong size dependence of particle migration in viscoelastic fluids, as indicated by expressions (1) and (2)^48,50,56,95,101–103^. Considering the relatively small lateral migration velocity of particles, which is generally 2−3 orders of magnitude slower than bulk flow^47^, dynamics of particle migration are readily observed in microfluidic devices due to their microscale characteristic lengths^56^. Factors that collectively determine particle migration and equilibration are discussed in this section, including the influence of fluid inertia, elasticity, rheology and channel geometry (see Box 3). Specifically, fluid inertia vs. elasticity, migration induced by the first normal stress difference (*N*_1_) vs. secondary flow induced by the second normal stress difference (*N*_2_), and elasticity vs. shear-thinning effect are identified as the three pairs of competitors that mainly govern the particle dynamics within microfluidic channel flows (Fig. 2). Thus, we next briefly discuss particle migration in the purely inertial flow before focusing on the two cases of viscoelastic fluid—migration in purely (inertialess) viscoelastic flows and migration in elasto-inertial flows.

Box 3 Phenomena that govern particle migration in microfluidic channels Forces responsible for particle migration in microfluidic channels stem from fluid inertia, elasticity and rheology. In Newtonian channel flows, neutrally buoyant particles are known to migrate to their preferential positions near the channel wall, driven by inertial force (*F*_i_)^34^. Shear-induced lift force (*F*_s_), wall-induced lift force (*F*_w_) and particle rotation-induced lift force (*F*_Ω_) are accounted for focusing of particles in inertial channel flows. In curved channels, Dean force (*F*_D_) due to secondary flow becomes significant. In viscoelastic flows, elastic force (*F*_e_) which mainly stems from the first normal stress difference (*N*_1_) dominates particle migration when inertia is negligible (Re ≪ 1)^48^. Similar to Dean force, secondary flows induced by second normal stress difference (*N*_2_) give rise to a drag force (*F*_*N*2_) that causes particles to follow the secondary flows in channel cross-sections^54,55^. Shear thinning of fluid rheology results in reduced viscosity of the fluid at high-shear rate, which increases inertial force and drives particles toward channel wall. We denote shear thinning effect on particle migration as *F*_st_. Shear thinning tends to defocus particles^51^. Characteristics of these forces on particle migration are listed in the table below.

**Table:** 

Source	Force or effect	Acting direction on particle migration
Fluid inertia	Shear-induced lift force (*F*_s_)	Up shear rate gradient (toward wall)
	Wall-induced lift force (*F*_w_)	Away from wall
	Dean force (*F*_D_)	Follow the secondary flows in curved channels
Fluid elasticity	First normal stress difference *N*_1_ (elastic force *F*_e_)	Down shear rate gradient (away from wall)
	Second normal stress difference *N*_2_	Follow the secondary flows in noncircular cross-sections
	(inducing drag force *F*_*N*__2_)	
Fluid rheology	Shear thinning	
	(inducing inertial force *F*_st_)	Toward wall

### Inertial migration in Newtonian flows

Neutrally buoyant particles suspended in a Newtonian fluid are known to migrate to their preferential positions near the channel wall. Spontaneous formation of the particle annulus near the pipe wall (Fig. 1e) was first observed by Segré and Silberberg^104,105^ more than 50 years ago. Extensive investigations have been implemented to explore the mechanism of this intriguing phenomenon^86,106–111^. Similar to the particle migration in viscoelastic fluids, wall confinement and the shear gradient are among the key factors that induce inertial lift force responsible for such particle migration in Newtonian channel flows (Fig. 2d).

**Fig. 3:**
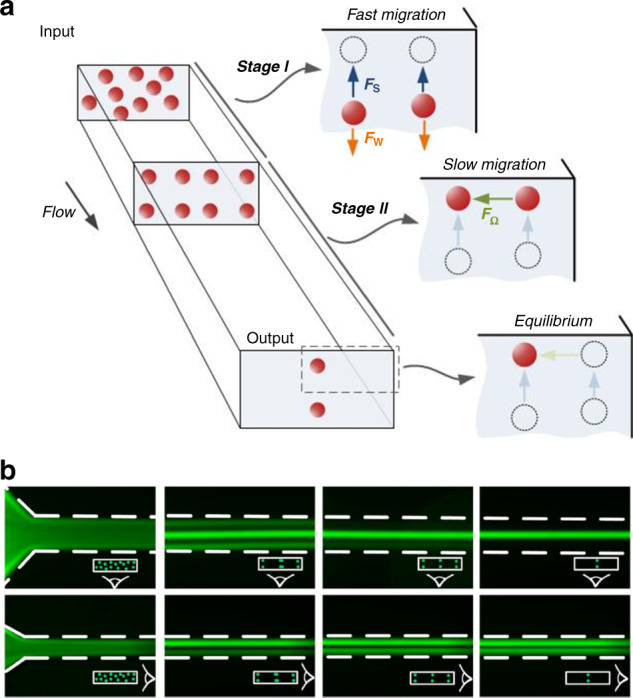
Two-stage migration of particles in inertia-dominant Newtonian flow and various focusing patterns in different inertial microchannels. **a** Fast migration of particles toward channel walls in the first stage dominated by shear-induced lift force (*F*_s_) and slow migration toward the stable positions in the second stage undergoing rotation-induced lift force (*F*_Ω_)^34^. Reproduced with permission from ref. ^34^. Copyright © Royal Society of Chemistry. **b** Fluorescent images of particle migration inside a rectangular channel validating the two-stage migration^128^. Reproduced with permissions from ref. ^128^. Copyright © AIP Publishing

Zhou and Papautsky^34^ described a two-stage model for particle migration at moderate Reynolds numbers (10 ≤ Re ≤ 100), which predicts particle dynamics in channels with various cross-sectional geometries (Fig. 3a, b). The shear-induced lift force (*F*_s_), wall-induced lift force (*F*_w_) and particle rotation-induced lift force (*F*_Ω_) account for the particle migration in the model. The balance between the first two forces is responsible for the formation of the Segré–Silberberg annulus in a pipe flow (Fig. 1e), and it is also the premise to substantiate the importance of the third force, which is typically an order of magnitude smaller than *F*_s_^34^. Particles undergoing *F*_s_ rapidly migrate toward the channel wall, where *F*_s_ is counteracted by the arising *F*_w_. In radially asymmetric channels, such as square and rectangular channels, the small rotation-induced lift force thereafter drives the particles toward the centers of each wall. As a result, four stable equilibrium positions can be observed in the square microchannel (Fig. 2d). Similarly, two stable positions can be formed in the centers of long walls in rectangular channels due to the differentiated velocity profiles along the two cross-sectional axes. The unstable positions in the corners of the square channel and those in the rectangular channel become preferential at high Re^112,113^. Particle focusing in various inertial channels is summarized in the review by Martel and Toner^39^. It is clear that the shear-induced lift force acts on the opposite direction of the elastic force, whereas the wall-induced lift force is in the same direction as the elastic force if they are present in a viscoelastic flow.

As inertial forces are highly size-dependent and applicable at high flow rates, they have been widely exploited for particle and cell manipulation in microfluidic devices^6,7,35,40,114–134^. Many applications in a variety of areas, such as size-selective inertial sorting^35,135^, high-throughput cell filtration^125,136^, microfluidic flow cytometry^137^ and isolation of rare cells (e.g., circulating tumor cells)^6,40,138^, have been proposed and demonstrated in the literature. Many other properties of inertial manipulation systems, including the channel layout, cross-section geometry, particle shape and deformability, have also been explored for improved performance and diverse applications. More details on inertial mechanisms and applications can be found in reviews by Martel et al.^39^, Amini et al.^38^ and Zhang et al.^42^.

### Particle migration in inertialess viscoelastic flows

At low Reynolds numbers (Re ≪ 1), inertial effects are decoupled, and it is possible to investigate particle migration under the influence of fluid elasticity alone (El ≫ 1). Indeed, the majority of the existing numerical simulation literature does this by assuming a negligible Reynolds number^47,82,84,93,100,139^. Experimentally, this is readily accessible, as viscoelastic fluids generally exhibit high viscosity, which yields small Reynolds numbers and limits fluid inertia effects. Under elastic force, particles migrate to the lower shear region of a microchannel cross-section, the axis of a circular channel or the centerplane in a wide rectangular channel^48,56,84,95,101,140^. Table 1 summarizes recent experimental studies that employ fluid elasticity for particle or cell manipulation within straight microfluidic channels. Leshansky et al.^48^ first observed the lateral migration and focusing of 8 µm diameter polystyrene particles in PVP solution (see Box 4 for common macromolecules used as elasticity enhancers in microfluidic systems and Box 5 for their rheological properties) toward the centerplane in their wide channel with 45 µm characteristic height (*β* = 18%). The focusing of particles becomes more pronounced with increased flow rate, which is in agreement with simulation results from planar Poiseuille viscoelastic flow^69,93^.

**Table 1:** Summary of recent viscoelastic microfluidics for passive focusing and separation in straight channels (units are converted, and values not shown in original work are calculated based on available parameters. Not all channel geometries or particle sizes in original references are included in this table, but the optimal numbers may be selected here. Shear rates were calculated using $\dot \gamma = 2Q/(WH^2)$ in rectangular channels and $\dot \gamma = 8Q/D^3$ in tubular channels (*D* is the diameter). All blockage ratios were calculated based on the available parameters, and only PS particles were used to calculate *β* whenever available. Asterisk marker (*) indicates that the value was used to calculate or was relevant to calculations of dimensionless numbers)

Mode	Dimensionless numbers				Sample flow rate (µl/h)	Shear rate (s^−1^)	Channel dimensions			Target particle		Carrier fluid or elasticity enhancer	Remarks	Ref.
	Re	Wi	El	*β*			Width (µm)	Height (µm)	Length (mm)	Type	Size (µm)			
Elasto	0.00158	N/A	N/A	0.11, 0.18	400–2000 100–2000	109–549 27.4–549	1000	45	20	PS	5, 8	PVP PAA	Particle *Re* 5 × 10^−5^	^48^
Elasto	~0.001	0.008–0.8	N/A	0.08	0.45–45	2.55–255	50 µm (microtube inner diameter)		45	PS	4	PVP*, PEO	Tube length 10 cm	^95^
Elasto	0.0037 0.016	0.36 0.4	98 25	0.11 0.10	60 534	267 297	50 100	50 100	50	PS	5.8 10	PVP	Optimal values	^101^
Elasto	0.11–0.33 0.0015–0.015	178–533 0.18–1.8	1618–1615 120	0.02–0.2	5–15 N/A	2.2 × 10^6^−6.7 × 10^4^ 20–200	5 30	5 10	40	PS DNA	0.1–1 0.69, 1.5	PEO	No focusing for 0.1 µm	^56^
Elasto	1.74	263	151	0.16	300	1.5 × 10^4^	30	50	10	PS	4.8	PEO	High sheath flow rate	^162^
Elasto	0.03–0.74	0.44–11	14.9	0.02–0.1	1200–30,000	31.4–786	300 (microtube inner diameter)		300	PS	7, 15, 30	PVP	Long channel	^51^
Elasto	0.035–2.3 0.018–7.1	7.9–507 4.0–1580	226–220 222–223	0.11 0.21	10–640 5–2000	57–3622 28−1.1 × 10^4^	50 µm (microtube inner diameter)		40	PS	5.8 10.5	DNA based	Microtube, Sheath for chromatography	^163^
Elasto	3.57 × 10^−6^−1.78 × 10^−3^	0.002-0.93	522–560	0.11, 0.23, 0.35	0.06–30	0.533–267	43 µm (microtube inner diameter)		30	PS RBCs	5, 10, 15 N/A	PVP	Two segments	^139^
Elasto-deformability	(1.2–9.9) × 10^–3^ (3.3–20) × 10^−3^	0.08–0.67 0.1–0.59	67.7 30.1	0.12	20–160 80–480	88.9–711 105–632	50 75	50 75	40	PS RBCs WBCs	6 N/A N/A	PVP	Deformability, separation purity >98%	^102^
PFF viscoelastic	0.3–0.6	N/A	N/A	0.22, 0.35, 0.21	20	890–1740	66	63	20	MV3, MCF, Hep G2	14 22 13	PBS/dextran PBS/RBC, whole blood	Varying Sheath flow	^149^
Elasto-inertial PFF	0.51−3.4	21.7−145	42.5*	0.06−0.4	15−100	1968−13,125	50	40*,100, 25	20	PS	3.1, 9.9	PEO	Sheath flow involved	^170^
Elasto-inertial PFF	0.2−1.35	3.81−25.4	18.8*	0.07−0.4	3−20	1120−7822	50	25*, 100, 40, 15	20	PS	4.18, 3.5−6	PEO	Peanut-shaped	^161^
Elasto-inertial	0.07−0.37	1.6−8.04	22.9-21.7	0.048, 0.12	40−200	178−1132	50	50	40	PS	2.4, 5.9	PEO*, PVP	3D in PEO	^50^
Elasto-inertial	0.5 0.81	12.84 12.7	25.68 15.7	0.02, 0.1	270	1200	50	50	25	PS RBCs	1, 5 8	PEO	Buffer for separation	^147^
Elasto-inertial	0.69 4.17	152 146.7	220 35.2	0.1−0.3	100 3000	1.4 × 10^4^ 6.7 × 10^3^	40 100	10 50	30 50	PS, *E. coli*, RBC PS, MCF, RBC	1, 3 ~1 8 5, 15 18 8	PEO	Separation achieved; large by side/small in center	^103^
Elasto- inertial	0.02−1.11 0.01−5.56	4.9−247 1.2−60	245−222 120−10	0.1, 0.17	1.2 × 10^3^−6 × 10^4^	24.7−1234	300	300	300	PS	30, 50	PVP PEO	Shear thinning	^96^
Elasto-inertial	0.21−8.55 0.07−9.97 0.06−10.16	2.43−97.7 0.45−63.7 0.29−52.7	11.6−11.4 6.43−6.39 5.2−4.8	0.04−0.2	60−4800 60−8400 60−10,800	267−21,333 133−18,666 89−16,000	50 100 150	50	40	PS	10, 5, 2	PEO*, PVP	Multiple stream focusing	^143^
Elasto-inertial	105−4422	17−566	0.16−0.13	0.01− 0.1	3.6 × 10^4^−1.2 × 10^6^	3.9 × 10^4^−1.3 × 10^6^	80	80	35	PS	1, 3, 6, 8	HA	WBC deformed	^151^
Elasto-inertial	2.5−34.5	1.5−12.2	0.35−0.60	0.08−0.4	3 × 10^3^−2.4 × 10^4^	5.9 × 10^3^−4.7 × 10^4^	25 70	250	40	PS, parasite, WBC	2, 10 1.5−2 9−15	HA	Two stages; separation achieved	^171^
Elasto-inertial	17−29	11−88	0.4−6.1	0.05−0.1	300	2.5 × 10^6^−5.5 × 10^6^	20	50	15	PS, bacteria, platelets	1−2	PEO	Viscoelastic flow as buffer	^166^

*HA* hyaluronic acid, *PVP* poly(vinyl pyrrolidone), *PEO* poly(ethylene oxide), *PAA* polyacrylamide, *RBC* red blood cell, *WBC* white blood cell, *PS* polystyrene particles, *FFF* field flow fractionation, *PFF* pinched flow fractionation, *N/A* not available or not able to be calculated

**Fig. 4:**
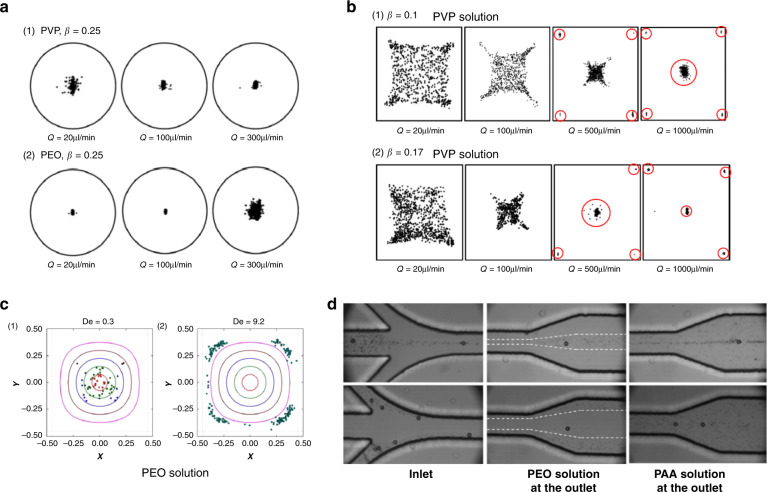
Particle dynamics in viscoelastic microchannels with negligible inertia. Particle focusing into the micropipe axis in PVP fluid with negligible inertia and particle defocusing in PEO solution due to the shear thinning effect at a high flow rate^51^. The channel length is 30 cm. Reproduced with permission from ref. ^51^. Copyright © Royal Society of Chemistry. **b** Four focusing positions near the corners in addition to channel axis in square microchannel and effect of particle blockage ratio on focusing in 8% PVP solution^96^. The channel length is 30 cm. Reproduced with permission from ref. ^96^. Copyright © AIP Publishing. **c** Four focusing positions near corners of a square channel due to strong shear thinning (1.6% wt PEO)^53^. The channel length is 8 cm. Reproduced with permission from ref. ^53^. Copyright © Springer Nature. **d** Effect of secondary flow on particle migration due to second normal stress difference in PAA solution (strong second normal stress difference *N*_2_) compared to the migration in PEO solution.^55^ Reproduced with permissions from ref. ^55^. Copyright © Springer Nature

Particles migrate toward the center of the circular channel cross-section due to fluid elasticity when the fluid inertia is negligible. D’Avino et al.^95^ and Seo et al.^51^ demonstrated particle focusing in a stable equilibrium position in the tube axis with Re ≪ 1 and *β* ≤ 10% (Fig. 1f). Enhanced focusing quality was found with an increasing flow rate (higher Wi or De), as shown in Fig. 4a. Romeo et al.^97^ proposed a dimensionless parameter Θ that accounts for the fluid rheology, flow rate, channel geometrical parameters and particle size. They found that particle focusing occurred within tubular microchannels when Θ > 1. Their prediction using Θ was valid in the limit of low elasticity (De < 0.05), negligible inertia (Re < 0.0005) and a certain blockage ratio (0.01 ≤ *β* ≤ 0.3)^97^.

Unlike the single focusing position in microtubes, multiple equilibrium positions exist within noncircular cross-sections of microchannels (Fig. 2). Since microfluidic channels with rectangular cross-sections are the most common, benefiting from soft-photolithography fabrication methods^141,142^, many researchers have probed particle migration dynamics in such channels^50,56,96,101–103,143–145^. As the driving force due to fluid elasticity (elastic force *F*_e_) is directed down the shear rate gradient^47^, particles are expected to migrate to the centerplane of a rectangular channel with an excessively low aspect ratio (AR, the ratio of cross-sectional height and width). Particles were found to concentrate on the middle plane in a slit with a small aspect ratio (AR = 0.045)^48^. As the AR increases (e.g., 0.25 < AR < 0.33), the focused band shrinks as a result of the altered shear rate gradient along the width^103,143^. Kim et al.^56^ used a similar rectangular channel for focusing DNA molecules (AR = 0.33 and Re ≪ 1).

In channels with square cross-sections (AR = 1), five distinct focusing positions emerge. Four equilibrium positions in the corners were observed in addition to the single position in the center of the cross-section (Fig. 2a)^50,56,96^. The presence of these additional positions is the result of the reduced shear rate in the corners^50^. Therefore, the migration direction depends on the initial positions of particles within the cross-section^54,100^. Without inertia, shear thinning and secondary flow effects, particles initially closer to the corners are attracted to the four positions, while other particles are driven by the elastic force to the position in the center. Interestingly, Del Giudice et al.^101^ observed only a single focusing position at the center in their square channel. The authors attributed this discrepancy to the surface property of their channel, but it is likely a result of the limited number of events recorded, as Seo et al.^96^ found a small number of particles in the corners (Fig. 4b) where more events were analyzed. Nevertheless, corner positions can be eliminated by introducing inertial forces, which will be discussed later^50^.

Contrasting the effect of fluid elasticity on particle migration, shear thinning of the fluid causes particle migration toward the channel wall in viscoelastic flows (Fig. 2b). Simulation results suggest that this effect becomes more significant when fluid elasticity is stronger (e.g., De > 2.5)^49,52^. The competition between shear thinning and fluid elasticity leads to an unstable separatrix between the channel centerline and wall^47,92^. With negligible inertia, particles initially located between the separatrix and channel wall migrate toward and equilibrate near the wall; particles on the other side of the separatrix are focused into the centerline. A strong shear-thinning effect moves the separatrix toward the channel centerline, leading to more particles migrating laterally to the wall^47,52^. Therefore, in square channels, the stable position in the center may disappear, leaving only the four positions in the corners when shear thinning is strong (Fig. 2b)^53^. The separatrix is close to the wall at weak shear thinning, and all particles migrate to the centerline^47,52^. As a result, particles may migrate bidirectionally under the influence of shear thinning, leading to particle focusing in both the channel centerline and the wall. This pattern of particle focusing was observed in a microtubular flow with a shear-thinning PEO solution (*β* = 0.16, De = 0.03, 1% wt PEO)^95^.

Shear thinning can also cause particle defocusing from the position in the channel center. As the flow rate increases, particles were found to be focused tighter and underwent stronger elastic forces in a viscoelastic flow without shear thinning (PVP solution in Fig. 4a). In contrast, particles were dispersed from the focusing position in the cross-section center (Fig. 4a) as an increased flow rate led to the onset of shear thinning in the PEO solution^51^. Similar observations were obtained in a square microchannel. When shear thinning was weak (De = 0.13), particles were focused mostly in the center position with some in the corner positions^53^. However, all particles were near corners, and no particle in the center was observed when shear thinning was very strong (De = 9.2 in PEO solution, as shown in Fig. 4c)^53^. Additionally, when inertia becomes comparable to elastic force, shear thinning leads to defocusing of particles from the channel centerline, and particles tend to move to the regions near the center of each wall^96^. For the commonly used PEO solution, shear thinning is weak when the PEO concentration is less than 2500 ppm^144^.

Similar to the interplay between shear thinning and fluid elasticity, migration due to *N*_*2*_-induced secondary flow can compete for elastic migration due to *N*_1_, leading to altered focusing dynamics within microchannels^54,55^. In noncircular channels, the second normal stress difference *N*_2_ is known to induce secondary flow perpendicular to the main flow direction^73,75^. For instance, in a square microchannel, a nonnegligible *N*_2_ induces eight recirculating vortices in the cross-section (Fig. 2c), and various recirculation secondary flows can also be generated in a rectangular microchannel depending on its aspect ratio^73,75^. The recirculation goes from the high-shear region to the low-shear region along the wall. Particles in the microchannels with noncircular cross-sections may follow the secondary flows undergoing drag force (Fig. 2c).

While elastic force focuses particles into equilibrium positions, *N*_2_-induced secondary flow tends to disperse particles. In a 3D simulation with negligible inertia, *N*_2_-induced secondary flow was found to affect the particle migration dynamics in a complex way depending on the blockage ratio and Deborah number^54^. For small particles (e.g., *β* = 0.1) and a large Deborah number (De = 2), the particle migration velocity due to the secondary flow surmounts the velocity owing to the elastic force stemming from *N*_1_. Consequently, particles follow the cross-sectional vortices, suggesting additional possible focusing positions in the eight vortices (Fig. 2c)^54,146^. This effect diminishes when De decreases (e.g., De < 0.5) or the particle size increases (e.g., *β* = 0.15)^54^. For a large particle, the migration due to elastic force is dominant and involves focusing into the centerline. These analytical results have been mostly validated by recent experiments in a rectangular microchannel (AR = 0.5) using PEO and polyacrylamide (PAA) solutions (Fig. 4d)^55^. *N*_2_ is not present in the PEO solution, but it is not negligible in the PAA solution. According to the confocal and microscopic images, smaller particles (*β* = 0.02) followed the secondary flow and could not be focused after introducing from the channel centerline in PAA solution. Conversely, larger particles (*β* = 0.2) remained stable in the centerline. Particle dispersion was not observed in the PEO solution under similar conditions. Based on this observation, separation of these two particles was also demonstrated by injecting the mixture slightly offset from the channel centerline in PAA solution^55^.

Box 4 Common macromolecules used as elasticity enhancers Macromolecules commonly used in microfluidic systems are listed in the table below. All of these materials exhibit shear thinning behavior, with PEO exhibiting the strongest shear-thinning^95^. PAA has a nonnegligible second normal stress difference^55^. Additionally, DNA sometimes can also be used as elasticity enhancer^163^. In terms of fluid rheology, either stronger or weaker, shear thinning can be achieved by varying amount of these macromolecules^53^. In fact, Boger fluids can also be approximated by mixing a small amount of high-molecular-weight polymer into a fluid with a relatively high viscosity^70^; such fluids show minimal change in viscosity with respect to shear rate in extended range but simultaneously exhibit distinct normal stresses, suggesting elastic behavior. Apart from mixing with water, these macromolecules are also commonly mixed into glycerol−water medium, which increases viscosity and thus reduces the Reynolds number (see Box 5 for details).

**Table:** 

Macromolecule	Abbreviation	Molecule weight (*M*_w_)	Typical concentration	Cells suspended
Poly(vinyl pyrrolidone)	PVP	3.6 × 10^5^ g/mol^48,50,51,95,96,101–103,139,176^	5−8% wt	hMSC, RBC, WBC
Poly(ethylene oxide)	PEO	2 × 10^6^ g/mol^50,56,147,161,162,170^	0.05−0.3% wt	MCF-7, RBC, *E. Coli*, DNA
		4 × 10^6^ g/mol^51,95,96,103^		
Polyacrylamide	PAA	N/A^48^	0.0045% wt	Microparticles
Hyaluronic acid	HA	1.65 × 10^6^ g/mol^151^	0.1% w/v	WBC
		0.357 × 10^6^ g/mol^151^		

*RBC* red blood cell, *WBC* white blood cell, *hMSC* human mesenchymal stem cell

Box 5 Rheological properties of common viscoelastic solutions used in microfluidics Polymer elasticity enhancers including PEO, PVP and PAA are usually mixed with water or glycerol. Glycerol is used to enhance the viscosity and thus reduce fluid inertia. Rheological properties of these prepared solutions are dependent on the concentration of the polymers used. Note that long time storage could reduce or eliminate shear thinning effect of some polymers. For example, little shear thinning was observed for PEO solution stored at room temperature without light for 3 months^103^. The table below shows the rheological properties of the aforementioned molecules with common concentrations at 20 °C. This table is reproduced with permission from ref. ^183^.

**Table:** 

Properties	PEO concentration (ppm)			PEO/glycerol (wt %)		PVP (wt %)	PAA (ppm)
	500	1000	2000	15	45	8	50
Density *ρ* (g/cm^3^)	1.0	1.0	1.0	1.03	1.10	1.05	1.0
Zero-shear viscosity *η*_0_ (mPa·s)	1.8	2.3	4.1	2.96	9.03	140	1.8
Effective relaxation time *λ*_e_ (ms)	4.3	6.8	10.6	11.0	24.0	2.3	10

PEO *M*_w_ = 2 × 10^6^ g/mol; PVP *M*_w_ = 3.6 × 10^5^ g/mol; PAA *M*_w_ = 18 × 10^6^ g/mol

### Elasto-inertial migration in viscoelastic flows

Thus far, we have discussed the effects of fluid inertia and elasticity on particle migration as mutually exclusive conditions. Complex migration dynamics can be expected when both are present in a viscoelastic fluid, and termed elasto-inertial migration (e.g., Re > 0.01 and Wi > 0). Coupled with the effects of shear thinning, particle blockage ratio and channel cross-sectional geometry, inertial and elastic forces act on particles synergistically in some scenarios, while antagonistically in others, dictating lateral migration of particles within microfluidic channels.

**Fig. 5:**
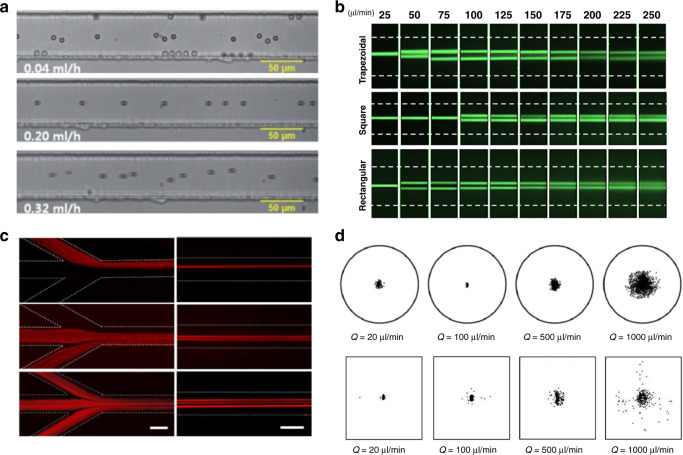
Particle dynamics in viscoelastic microfluidics with effects of inertia and shear thinning. **a** Inertial force necessary to eliminate the four corner positions in the square channel. A single stream of particles was observed at an increased flow rate where inertial force was sufficient to repel particles from four corners^50^. The channel length is 4 cm. Reproduced with permission from ref. ^50^. Copyright © Royal Society of Chemistry. **b** One and two streams observed in rectangular, square and trapezoid channels when both elastic and inertial effects are present^145^. The channel length is 5 cm. Reproduced with permission from ref. ^145^. Copyright © AIP Publishing. **c** One, two and three focused streams observed in the rectangular channel^144^. The channel length is 4 cm. Reproduced with permission from ref. ^144^. Copyright © 2017, American Chemical Society. **d** Defocusing of particles observed in both circular micropipes (*β* = 0.1)^51^ and square microchannels (*β* = 0.17)^96^ when the shear thinning effect was strong (1% wt PEO). The channel length is 30 cm. Reproduced with permission from ref. ^96^. Copyright © AIP Publishing

The synergistic effect of fluid elasticity and inertia promotes particle lateral migration necessary to achieve a single position focusing in square microchannels (Fig. 2e)^50,96,147^. Although inertia is negligible at a small Reynolds number (Re ≪ 1), its effect on particle migration becomes pronounced and serves to eliminate the otherwise stable positions in the corners of a square microchannel when Re is increased to approximately unity (e.g., 0.01 < Re < 10). In particular, the wall-induced lift force (*F*_w_) displaces particles away from the corner positions. Subsequently, elastic force (*F*_e_) drives them toward the channel centerline where the only stable position exists (Fig. 2e). Although the shear-induced lift force acts in the opposite direction of *F*_w_ and *F*_e_, the latter two are dominant when Re is small^50^. Considering the strong size dependence of the wall-induced lift force ($F_{\mathrm w} \propto a^6$) near the wall region^148^ and the predominant elastic force at a high elasticity number (e.g., El = 21), large particles can be focused into the cross-section center undergoing the synergetic interaction of the two forces, which has been demonstrated in microfluidic devices (Fig. 5a)^50,147^.

Unlike the synergetic interaction of *F*_w_ and *F*_e_, the shear-induced lift force (*F*_s_) counteracts the elastic force (*F*_e_) when particles are away from the channel wall. This competing effect is responsible for the varying focusing positions observed in the centerplane of rectangular channels (AR < 1)^103,144,145^. Dissimilar to a wide band in the centerplane observed in inertialess viscoelastic flow, the band evolves into 1, 2 and 3 focused streams depending on the blockage ratio and channel AR when inertia is not negligible in PEO solutions (Fig. 5b, c)^103,144,145^. The shear-induced lift force drives particles in the centerplane horizontally toward the two sidewalls, where it is balanced by a horizontal elastic force due to the large shear rate gradient. The balance of these two forces gives rise to two focused positions in the centerplane but near sidewalls (Fig. 2f). Increasing the fluid elasticity (larger El) brings the two focused positions closer to each other^144^. A further increase in El leads to the merging of the two positions into a single position in the center^144^. The mechanism of the presence of three focused streams remains to be determined.

Although the PEO solution and its shear-thinning effect are commonly used to eliminate corner focusing positions in square channels, this effect leads to particle defocusing in the center position at a high-shear rate (Fig. 5d). Seo et al.^51,96^ observed that particles (*β* = 0.05 and *β* = 0.1) started to defocus in 1% (wt) PEO solution when the flow rate exceeded 100 μL/min in their 300 μm diameter microtube ($\dot \gamma > 157\,{\mathrm{s}}^{ - 1}$). This is due to shear thinning becoming more pronounced when the shear rate is larger^144^, which displaces particles toward the wall in viscoelastic flows, as already discussed^52,146^. Shear thinning reduces viscosity (*η* ≈ 0.33 pa s at $\dot \gamma = 10\,{\mathrm{s}}^{ - 1}$, and *η* ≈ 0.42 pa s at $\dot \gamma = 1000\,{\mathrm{s}}^{ - 1}$)^51,96^, leading to increased Re. Nonnegligible inertial forces at higher Re start competing with elastic force at high-shear rate. For the PEO solution, shear thinning is weak when the concentration is below 0.25% (wt) in water but becomes significant when the concentration is higher^144^. Furthermore, when inertia is relevant, strong shear thinning at a high flow rate reduces fluid viscosity and thus effectively amplifies the Reynolds number, which results in an increased inertial force (*F*_s_) that drives particles away from the centerline.

The particle blockage ratio also influences the focusing pattern in a square channel^96^. Larger particles (*β* = 0.17 and *β* = 0.25) were found to show better resistance to the wall-pointed effect of shear thinning than smaller particles (*β* = 0.1)^51,96^. Interestingly, the focusing pattern observed for the smaller particles (*β* = 0.1) at a low flow rate is close to the combination of inertial focusing in a Newtonian fluid and elastic focusing with shear thinning, with four positions close to the centers of each wall and one position in the channel centerline^96^. The results from multiple simulations^52,93^ also suggest different migration dynamics for varying blockage ratios. However, the prediction that particles always migrate to the channel wall when the blockage ratio exceeds a critical value (*β* = 0.25 in the work by Huang et al.^52^) differs from experimental results that showed no particle focusing near the wall when *β* = 0.25 ^51^. Similarly, particles^139^ and cells^149^ were found to migrate toward the channel centerline even when *β* = 0.35. Simulation results by Villone et al.^93^ suggest that the critical value should be ~0.75.

When the inertia and fluid elasticity are comparable (El ~ 1), elastic force is found to be dominant in particle focusing dynamics. Recent 2D simulations^150^ suggest that particle migration is dominated by fluid viscoelasticity in this case and that the inertia is negligible. The domination of elastic force has been confirmed experimentally by Lim et al.^151^ in a weakly viscoelastic flow at El ≈ 0.15. Particles with a diameter of 8 µm were focused at the centerline of a square microchannel in a hyaluronic (HA) solution at Re up to 4422 (Wi = 556). Nonetheless, the absence of particles in the corners implies that inertial forces are not negligible. Similar results can be found in the work by Del Giudice et al.^53^, where particle migration toward the centerline was observed at both El ≈ 0.4 and El ≈ 40. These results, along with simulations, suggest that particle migration is dominated by elastic force when ${\cal{O}}\left( {{\mathrm {El}}} \right) = 1$. These results show the inability of using the elasticity number alone for the prediction of particle migration dynamics. The 2D simulations^150^ suggest that Wi has to be at least two orders of magnitude smaller than Re for the inertial forces to be competitive.

In this section, we have seen how particles migrate in purely (inertialess) viscoelastic flows and elasto-inertial flows. In an inertialess (Re ≪ 1) viscoelastic flow, without the effects of shear thinning and secondary flow, particles are driven to their stable focusing positions within channel cross-sections by elastic force, which is attributed mainly to the first normal stress difference (*N*_1_). The migration can be sped up either by increasing the fluidic elasticity or by a larger particle blockage ratio. The number of equilibrium positions varies with the cross-sectional geometry, with a single position in the channel centerline in circular channels, an additional four positions located in the corners of square channels, and various positions in the centerplane of rectangular channels. Additionally, shear thinning at a high-shear rate reverses the particle migration direction and displaces the particles toward the four corners of square channels, and the existence of secondary flow due to the second normal stress difference *N*_2_ causes particle migration following the recirculating flow orthogonal to the main flow, disrupting the 5-position focusing pattern. Smaller particles are found to be more susceptible to secondary flow.

Finally, the competition between the inertial and elastic forces in viscoelastic flow dictates particle focusing dynamics when inertia is not negligible (e.g., Re > 0.01). While the focusing dynamics remain the same as the inertialess viscoelastic flow in circular channels, a single focusing position in the center of square cross-sections is achieved with corner positions eliminated by wall-induced lift force at increased Re. The competition of shear-induced lift force and elastic force in the horizontal direction leads to varying focusing positions observed in low-AR rectangular microchannels^103,144,145^. Surprisingly, fluid elasticity remains dominant in determining particle migration dynamics even when inertial and elastic forces are comparable (${\cal{O}}\left( {{\mathrm {El}}} \right) = 1$). Nevertheless, the effect of inertial force is evident under this condition. Additionally, the shear-thinning effect and particle blockage ratio can modify migration dynamics. As a result, particles in viscoelastic flow show complex behavior that is dependent on the interactions among inertia, elasticity (*N*_1_), shear thinning, secondary flow (*N*_2_), particle blockage ratio and channel cross-sectional geometry. Two additional factors that contribute here are the overall channel geometry and the physical properties of particles, which we will discuss in the next section.

## Channel geometry and particle physical properties

In addition to the cross-sectional geometry, the overall geometric layout of the microfluidic channel can significantly impact the particle migration dynamics. In Newtonian flow, the curvature of a microchannel is known to induce a pair of counterrotating secondary flows in its cross-section^115,124^. In a rectangular spiral channel, such recirculation reduces the number of focusing positions to only one, typically near the inner wall where the shear-induced lift force (*F*_s_) is balanced with the Dean drag force (*F*_D_)^123,124^. Due to the high-throughput nature of spiral microchannels, they have been used for the isolation of rare cells^40,152^.

**Fig. 6:**
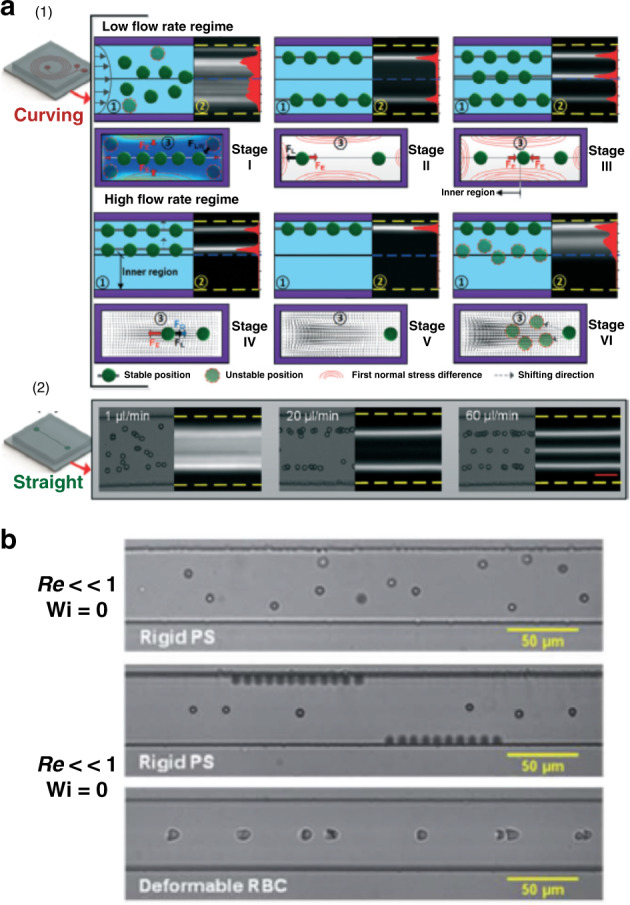
Effects of channel geometry and particle deformability on lateral migration and focusing. **a** Various focused streams observed both in spiral and straight rectangular microchannels when inertia was relevant^154^. Reproduced with permission from ref. ^154^. Copyright © Royal Society of Chemistry. **b** Single position focusing of red blood cells attributed to the interaction of the elastic force and deformability-induced lift force^102^. Reproduced with permission from ref. ^102^. Copyright © Royal Society of Chemistry

Similarly, the curvature of the channel is found to alter the focusing dynamics of particles in viscoelastic flows. Lee et al.^153^ showed that the strong Dean drag was counteracted by the elastic force instead of the shear-induced lift force, leading to a single focusing position near the center of the outer wall. This focusing position is on the opposite side of Newtonian flow focusing. Another work^154^ confirmed the distinctive equilibrium position and showed in detail the progressive evolution of focusing positions as the flow rate increased (Fig. 6a). Using a double-spiral channel, focusing of 100 nm diameter particles was achieved recently^155^. Instead of achieving single-position focusing in a spiral channel^50^, Cha et al.^156^ introduced expansions to the straight channel. The expansions lead to the formation of curved streamlines, which induce Hoop stress directed toward the channel center^71^, leading to single-position focusing in an inertialess viscoelastic flow. A similar effect of triangular expansions was observed in PEO solution^157^, and separation based on cell sizes was achieved recently^158^.

Particle physical properties such as deformability and shape can also significantly impact migration dynamics in viscoelastic flows. These properties are known markers for differential manipulation within microfluidic channels in Newtonian fluids^159,160^. Particle deformability in viscoelastic flow has been demonstrated in square microchannels using red blood cells (RBCs), as shown in Fig. 6b^102^. Deformable cells were found to experience an additional force that drives them away from the corners, leading to 3D focusing in an inertialess flow and thus better separation performance than in inertial flows (enrichment ratio 336 vs. 69)^102^. In terms of particle shape, peanut-shaped particles were found to migrate closer to the channel sidewalls than spherical particles in an elasto-inertial pinched flow fractionation (ei-PFF) device^161^. Other flow configurations, such as sheath flow, can be used to help manipulate particle migration in viscoelastic flow^147,162,163^. Ultimately, both the deformability and the shape of particles lead to distinct migration dynamics in viscoelastic flows since both cause changes in the effective particle size.

## Numerical simulations

**Fig. 7:**
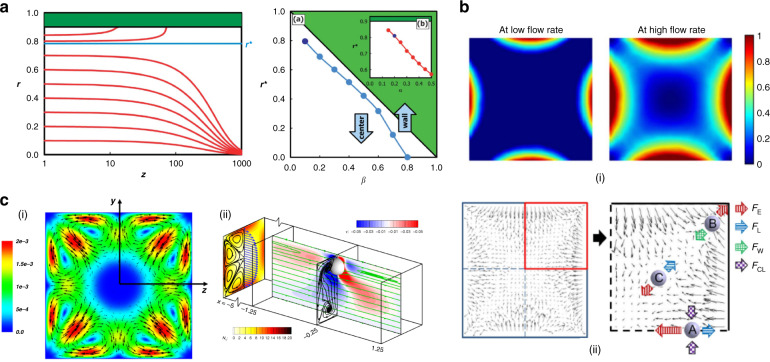
Numerical simulations predicting particle migration and focusing in viscoelastic channel flows. **a** Giesekus model used to predict particle downstream trajectories (red curves) and the dependence of migration direction on particle blockage ratio (*β*) in a micropipe flow^95^. Reproduced with permission from ref. ^95^. Copyright © Royal Society of Chemistry. **b** COMSOL Multiphysics® was used to predict the distribution of the first normal stress difference (*N*_1_) and the vector of lateral force in a square channel cross-section^96^. Reproduced with permission from ref. ^96^. Copyright © AIP Publishing. **c** Simulations of the effect of secondary flow induced by the second normal stress difference (*N*_2_) on particle migration in square microchannels using the Giesekus model^54,146^. Reproduced with permission from refs. ^54,146^. Copyright © Elsevier and copyright © Cambridge University Press, respectively

Numerical simulations are frequently used in conjunction with experimental studies when investigating particle migration in viscoelastic flow. Numerical simulations offer a convenient way to predict and visualize particle migration in 3D, which helps guide experimental investigations. The most common models used for numerically simulating viscoelastic flow include the Oldroyd-B, Giesekus and Phan-Thien Tanner models^47,52,96,146^. The upper-convected Maxwell (UCM) model, or Oldroyd-B model, predicts the first normal stress difference (*N*_1_) on particle migration without considering shear thinning (constant viscosity). The Giesekus and Phan-Thien Tanner models consider the shear thinning effect, with the former also predicting the second normal stress difference (*N*_2_)^47,54^. These models predict the motions of particles in viscoelastic flows, which help decipher the complex and often competing effects of fluid rheology on migration, such as the outward migration caused by shear thinning and inward migration due to *N*_1_ (Fig. 7a)^52,54^. However, significant skills in computational modeling are required. Some experimental researchers used the commercially available software COMSOL Multiphysics® to simulate the distribution of *N*_1_ and force vectors in the cross-sections of microfluidic channels (Fig. 7b)^50,96^. Particle motions and trajectories are not available using COMSOL Multiphysics®.

Motions of particles suspended in simple shear, planar Poiseuille and microfluidic channel flows have been simulated using the three models for deciphering the effects of *N*_1_, *N*_2_, shear thinning, and inertia. Many existing simulations report on particle migration in simple shear and Poiseuille flows, either in 2D or in 3D. Particles were found to migrate toward the closest wall in simple shear flow^82,83,140^. In planar Poiseuille flows, simulation results were consistent with experimental observations that particles migrate toward the centerline when fluid elasticity was dominant and inertia was negligible^93,150^. Those also extensively examined in Poiseuille flows include the competitions between inertial and elastic forces^93,150^ and between elastic force and shear thinning^49,52^. In channel flows, simulations were implemented in straight microchannels with circular^95^ or more commonly square^50,53,54,145,146,164^ cross-sections to investigate the effects of elasticity, inertia, shear thinning and secondary flow induced by *N*_2_. Simulation results found that secondary flow and shear thinning tend to move particles away from the channel center^54,146^. Additionally, recent simulation work^165^ suggests that particle deformability contrasts with migration due to elasticity (Fig. 7c). Simulation results are common compared with experimental observations, and discrepancies can sometimes be noted^51,139,149^. The discrepancies may be attributed to the limited channel length in the experiments and the deformation of the channel cross-section under fluid pressure.

## Applications of viscoelastic microfluidics

**Fig. 8:**
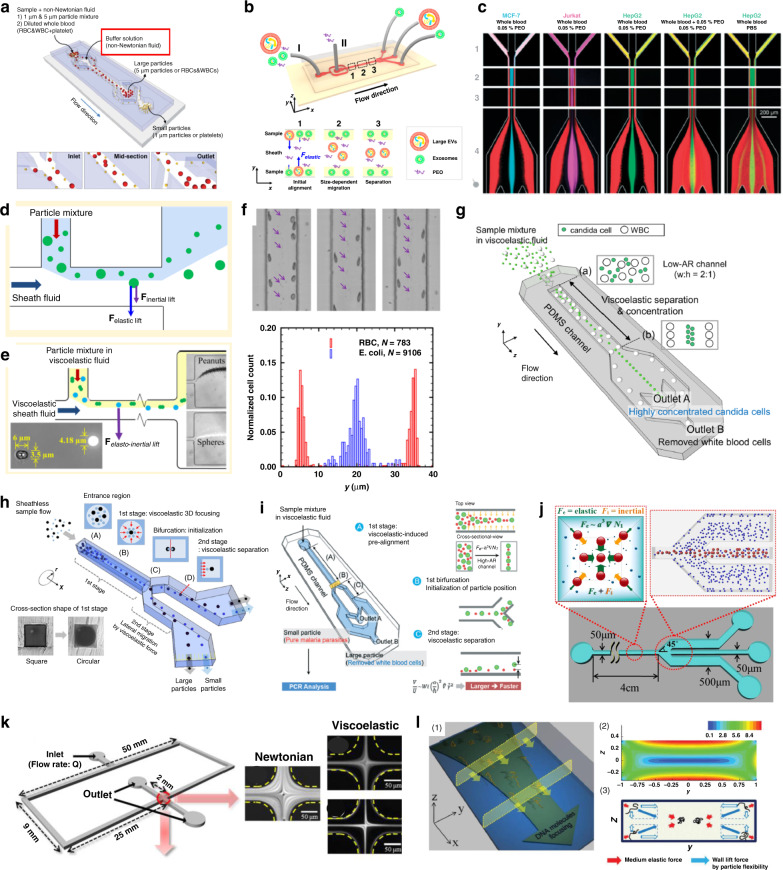
Recent applications of viscoelastic microfluidics. **a** Size-selective particle, cell and exosome separations based on elastic force achieved in microchannels with sheath flow in the center^147^. Reproduced with permission from ref. ^147^. Copyright © Royal Society of Chemistry. **b** Viscoelastic coflow for exosome separation^57^. Reproduced with permission from ref. ^57^. Copyright © 2017, American Chemical Society. **c** Tumor cell-line cell separation from blood using PEO core flow^169^. Reproduced with permission from ref. ^169^. Copyright © Royal Society of Chemistry. **d** Combination of viscoelasticity and inertia for pinched flow fractionation (eiPFF)^170^. Reproduced with permission from ref. ^170^. Copyright © 2015, American Chemical Society. **e** Shape-based separation of peanut particles in a viscoelastic PFF device^161^. **f** Sheathless separation of RBCs and *E. coli* in viscoelastic fluid flowing in a rectangular microchannel^103^. Reproduced with permission from ref. ^161^. Copyright © 2015, American Chemical Society. **g** Separation of fungus from blood in a rectangular viscoelastic channel^174^. Reproduced under a Creative Commons Attribution 4.0. **h** Microchannels consisting of two segments for sheathless particle separation^139^. Reproduced with permission from ref. ^139^. Copyright © Elsevier. **i** Separation of malaria parasites from WBCs in a two-segment channel^171^. Reproduced with permission from ref. ^171^. Copyright © Royal Society of Chemistry. **j** Filtration of particles in a square microchannel using both elastic and inertial forces^175^. Reproduced with permission from ref. ^175^. Copyright © Elsevier. **k** Single-stream focusing of cells in viscoelastic microflow used for measurements and monitoring of cell deformability^176^. Reproduced with permission from ref. ^176^. Copyright © 2012, American Chemical Society. **l** Focusing of DNA molecules in a low-AR rectangular microchannel based on the elastic force and flexibility-induced lift force^56^. Reproduced with permission from ref. ^56^. Copyright © 2012, American Chemical Society

Currently, the main applications of viscoelastic microfluidics are in the focusing and separation of particles and cells in microfluidic devices. Compared to inertial microfluidics, which have been widely used for these applications^42^, particle manipulation in viscoelastic flows is particularly intriguing due to its advantages, including focusing of submicron particles, 3D focusing and wide dynamic range of flow rate^50^. While inertial microfluidics are effective in manipulating particles or cells a few microns or above in size (*a* > 3 μm), viscoelastic microfluidics have shown reliable performance in focusing and separating smaller particles (1 μm or below), such as bacteria^166^, exosomes^57^, and DNA^56^. To date, most literature related to viscoelastic flow in microfluidic channels lies in the exploration of particle focusing and the underlying mechanisms. While single position focusing holds great potential for applications such as flow cytometry^167^, it might be deemed less useful in separation applications because it is not easy to differentiate particles due to a single focusing position. Examples of recent applications of viscoelastic microfluidics are highlighted in Fig. 8.

Viscoelastic microfluidic devices are effective in sorting particles and cells ranging from tens of nanometers to tens of microns in size. The earliest such platform was proposed by Nam et al.^147^ who used buffer solution in the middle of a microchannel flanked by two sample flows (Fig. 8a). Due to the size-dependent migration toward the stable positions in the centerline (see expression (1)), separation of particles and blood cells was readily achieved with efficiency and purity both as high as 99%^147^. Since then, the same flow configuration has been used to separate exosomes (30−200 nm) from other EVs (Fig. 8b)^57^, bacteria (1 μm) from platelets^166^ and leukocytes^168^, and circulating tumor cells (CTCs) from blood (Fig. 8c)^169^. Particle migration due to viscoelasticity has also been coupled with pinched flow fractionation (ei-PFF) to achieve easy separation (Fig. 8d, e)^161,170^. However, the throughput of both approaches was mediocre (<90 µL/h).

Sheathless separation of particles can be achieved using either rectangular channels (Fig. 8f, g) or two-segment assembly of microchannels (Fig. 8h)^139^. Rectangular channels are capable of positioning particles or cells into different lateral positions based on size as a result of the interaction of inertial and elastic forces^103^. The two-segment configuration was modified to improve the throughput (Fig. 8i)^171^. Using HA instead of PEO solution, the throughput was increased to 400 µL/min, while the separation efficiency and purity remained competitive. The separation of malaria parasites from white blood cells (WBCs) was successfully demonstrated in this device, with a 94% recovery rate for parasites and 99% recovery rate for WBCs. The purity reached 99% for both parasites and WBCs. Size-selective separation of particles was also reported using ferrofluid for magnetophoresis after an initial 3D focusing in a similar two-segment channel^172^. Recently, Liu et al.^103^ showed the high-profile separation of MCF-7 cells from red blood cells (RBCs) and separation of *E. coli* from RBCs in low-AR rectangular channels (Fig. 8f). A similar design was used for the separation of microalgae and bacteria^173^ and for the filtration of fungi from WBCs (Fig. 8g)^174^. Additionally, particle separation was also reported in square microchannels by tuning the inertial forces^175^ and by an external magnetic field (negative magnetophoresis of the viscoelastic ferrofluid)^172^ (Fig. 8j). Separation performance in viscoelastic fluids is generally excellent in terms of efficiency and purity, although the throughput remains mediocre.

Other applications, including measurement of cell deformability and orientation control, have also been demonstrated in viscoelastic microchannels. Cell deformability itself can be a biomarker for cell sorting since it gives rise to the additional lift force that can eliminate the corner focusing positions, promoting cell focusing in the channel centerline^102^. Cha et al.^176^ used this phenomenon for measurements of cell deformability (Fig. 8k). A cross-slot channel was designed after the square focusing channel to allow cells to be stretched undergoing extensional flow in viscoelastic fluids. Changes in RBC deformability due to heat shocks were measured, and a decrease in deformability in human mesenchymal stem cells (hMSCs) due to nutrient starvation was monitored in their device. While the flow rate is far from impressive (160 µL/h), such an approach does not require a sophisticated design of channels and avoids the adverse influence of multiple focusing positions in inertial microfluidic channels. Other applications include focusing on macromolecules (e.g., DNA^56^), which is otherwise challenging in Newtonian inertial devices (Fig. 8l), and orientation control of nonspherical bioparticles (e.g., RBCs^177,178^). Additionally, relaxation time measurement of viscoelastic fluids was proposed based on particle focusing in a straight channel^79^ and the onset of instability in a serpentine channel^179^.

## Concluding remarks and perspectives

The understanding of complex particle dynamics in viscoelastic flows has dramatically improved in recent years. The well-established knowledge of viscoelastic materials has promoted the advancement of research on particle interactions with surrounding fluids. For many years, investigations of such interactions had to rely mostly on numerical simulations, while experimental investigations were limited to simple shear or concentric Couette flows and planar Poiseuille flows. The existence of imbalanced normal stress differences not only results in distinct phenomena in viscoelastic fluids, such as the Weissenberg rod climbing effect, but also leads to the lateral migration of suspended particles^47^.

Applying microfluidic techniques to investigations of viscoelastic flows has extended the research frontier into the microscale as well as into the probing of the broad spectrum of particle dynamics. Advancements in microfabrication readily provide access to microchannels with a high ratio of channel length to characteristic length (e.g., *D*_h_). These microchannels enable observation of full particle dynamics on a short time scale since the lateral migration is typically two to three orders of magnitude smaller than the downstream velocity^47^. The microscale characteristic length also permits an easy decoupling of the effect of fluid inertia (Re ≪ 1) to allow the inquiry of the sole effect of fluid viscoelasticity on particle dynamics. Elastic force stemming mainly from the first normal stress difference *N*_1_, the effect of fluid shear thinning, and the secondary flow induced by the second normal stress difference *N*_2_ collectively govern particle migration behavior and its equilibrium positions within channel cross-sections in inertialess flows (Re ≪ 1). As the Reynolds number increases, inertial forces become relevant and interact with the above factors, giving rise to distinct focusing dynamics of particles, such as single-position (3D) focusing in square channels^50^ and multiple-stream focusing in rectangular channels^143,144^. More intricate interactions and competitions can occur when channel geometry and particle properties (e.g., deformability) are involved. Nevertheless, constant effort in experimental and numerical studies has further unraveled the migration physics in viscoelastic flows.

Despite tremendous progress, a number of questions remain unanswered. First, although the elasticity number is commonly used for assessing the relative importance of fluid elasticity and inertia, this number alone is not sufficient. Recent experimental results^53,151^ showed particle focusing in the centers of square channels when inertial and elastic forces were thought to be comparable as El ~ 1. Inertial forces exhibited only auxiliary influence to eliminate the corner focusing positions, and the particle focusing pattern remained unchanged even when El < 1. This echoes a 2D direct simulation^150^ that suggests that “El < 0.01” is necessary for the inertial effect to be dominant in the viscoelastic flow. Currently, no work has been reported with experiments in such regimes to determine a more accurate criterion for assessing the relative importance of fluid elasticity and inertia on particle migration. Table 1 shows that the smallest El reported is 0.13. An enlarged channel characteristic length might be helpful to achieve a small El in viscoelastic microchannels.

Second, more work is still necessary to elucidate the shear-thinning effect in viscoelastic flow with nonnegligible inertia. According to the simulations^52^, most particles migrate to the core area of the channel centerline, while others form long chains and move along the walls. Although particle defocusing from the centerline was evident at a high-shear rate, the formation of long chains and particle annuli near walls was not observed in experiments^96^.

Third, the particle blockage ratio shows complex and sometimes contradictory effects on particle migration dynamics and focusing positions. While the elastic force is strongly dependent on particle size, direct simulations suggest the opposing effect of the blockage ratio: particle migration toward the centerline is generally dominated by the elastic force, but migration can reverse when the particle size exceeds a critical value^49,93^. Reversed migration due to large particle size has not been reported.

Furthermore, focusing dynamics in rectangular channels remains not fully understood, especially when inertia is relevant. Rectangular channels are the most popular channels for applications such as cell sorting. Laterally distinctive focusing positions for particles of different sizes enabled easy separation of cells and *E. coli* in a straight channel^103^. However, the force competitions that lead to multiple focusing positions in the centerplane remain unclear. While the balance between inertial force (shear-induced lift force) and elastic force explains the formation of two streams straddling the centerline, the current understanding does not explain the presence of a focusing position at the center when multiple positions exist.

Finally, many combinations of fluid composition, flow conditions, and microchannel geometry remain unexplored. The most commonly used channel geometry is a rectangular cross-section, and viscoelastic flows are frequently investigated in these microchannels either with or without inertia. Particle migration in viscoelastic flow with strong shear thinning but negligible inertia has been examined in square straight microchannels only^53^. Other geometries, such as circular and triangular microchannels, receive even less attention, as fabrication of such microchannels is not straightforward. Nonetheless, microchannels with triangular cross-sections can be interesting to explore for viscoelastic flow, as unexpected focusing positions of particles were found in Newtonian flows^43,180^. Similarly, the migration dynamics of particles in spiral channels are not fully explored in viscoelastic flow. When inertia is relevant, the focusing position of particles is generally near the outer wall instead of near the inner wall in inertial microfluidics. Two other conditions (inertialess and coupled with strong shear thinning) were not investigated, possibly due to negligible Dean vortices. Nevertheless, the change in the velocity profile under such conditions is likely to influence particle migration.

In terms of applications, focusing and separation are the main themes so far. Viscoelastic microfluidics has shown excellent performance, particularly in the separation of small particles and in precise spatial manipulation, such as 3D focusing, as well as a broad dynamic range suitable for various applications. The existing literature suggests the high (e.g., ≥99%) efficiency and purity of such devices. Nonetheless, the throughput of these systems is typically much lower than that of their inertial counterparts using Newtonian fluids. Owing to the high viscosity, viscoelastic microchannels require high driving pressures but generally manifest smaller Reynolds numbers. Based on the elasticity number, the practical way to improve the throughput seems to be tuning the fluid rheology and characteristic length, as shown in recent works^51,151^.

The current enthusiastic investigations into the viscoelastic manipulation of particles in microchannels lead to a new promising area of microfluidics, viscoelastic microfluidics. Considering the viscoelastic nature of many biological samples, such as blood, viscoelastic microfluidics suggest the possibility of handling biosamples with minimal preparation. More work into the particle dynamics in complex conditions will be imperative to achieve such perspective as sample dilution is currently required to avoid particle−particle interactions. Despite the promise in processing biosamples, a few disadvantages of this method for particle or cell separation need further attention. These include the low throughput despite the dynamic range being wide (theoretically), and the typical requirement of elasticity enhancers, which can contaminate samples. Due to the high viscosity of some viscoelastic flows, this method also necessitates a high pressure drop inside microfluidic channels, which can cause device failure in practice.

Another exciting advantage of viscoelastic microfluidics is its capability of handling submicrometer particles (e.g., exosomes) and macromolecules, such as DNA and proteins. Manipulation of EVs and macromolecules without sophisticated and time-consuming labeling can be ideal and is in high demand. Inertial microfluidics typically works well on the cell-size scale (*a* > 3 μm), but viscoelastic microfluidics empowers the manipulation of smaller sizes down to nanometers^57,155^. For instance, the secondary flow induced by the second normal stress difference is more effective in controlling dynamics of small particles rather than large ones.
